# Dengue virus serotype 2 infection alters midgut and carcass gene expression in the Asian tiger mosquito, *Aedes albopictus*

**DOI:** 10.1371/journal.pone.0171345

**Published:** 2017-02-02

**Authors:** Hitoshi Tsujimoto, Kathryn A. Hanley, Anitha Sundararajan, Nicholas P. Devitt, Faye D. Schilkey, Immo A. Hansen

**Affiliations:** 1 Department of Biology, New Mexico State University, Las Cruces, New Mexico, United States of America; 2 NM-INBRE Sequencing and Bioinformatics Core, National Center for Genome Resources, Santa Fe, New Mexico, United States of America; University of Texas Medical Branch, UNITED STATES

## Abstract

**Background:**

The Asian tiger mosquito, *Aedes albopictus* is currently an important vector for dengue, chikungunya and Zika virus, and its role in transmission of arthropod-borne viruses (arboviruses) may increase in the future due to its ability to colonize temperate regions. In contrast to *Aedes aegypti*, the dominant vector of dengue, chikungunya and Zika virus, genetic responses of *Ae*. *albopictus* upon infection with an arbovirus are not well characterized. Here we present a study of the changes in transcript expression in *Ae*. *albopictus* exposed to dengue virus serotype 2 via feeding on an artificial bloodmeal.

**Methodology/Principal findings:**

We isolated midguts and midgut-free carcasses of *Ae*. *albopictus* fed on bloodmeals containing dengue virus as well as controls fed on virus-free control meals at day 1 and day 5 post-feeding. We confirmed infection of midguts from mosquitoes sampled on day 5 post-feeding via RT-PCR. RNAseq analysis revealed dynamic modulation of the expression of several putative immunity and dengue virus-responsive genes, some of whose expression was verified by qRT-PCR. For example, a serine protease gene was up-regulated in the midgut at 1 day post infection, which may potentially enhance mosquito susceptibility to dengue infection, while 14 leucine-rich repeat genes, previously shown to be involved in mosquito antiviral defenses, were down-regulated in the carcass at 5 days post infection. The number of significantly modulated genes decreased over time in midguts and increased in carcasses.

**Conclusion/Significance:**

Dengue virus exposure results in the modulation of genes in a time- and site-specific manner. Previous literature on the interaction between mosquitoes and mosquito-borne pathogens suggests that most of the changes that occurred in *Ae*. *albopictus* exposed to DENV would favor virus infection. Many genes identified in this study warrant further characterization to understand their role in viral manipulation of and antiviral response of *Ae*. *albopictus*.

## Introduction

The Asian tiger mosquito, *Aedes albopictus* originated in Asia but has invaded most of the tropical and subtropical areas of the world [1]. It now occurs many countries in Europe, Africa, South America, the Caribbean, and North America, including 32 states in the continental United States [2] and Hawaii [3]. Further global expansion is expected in connection with global climate change and urbanization [4]. *Ae*. *albopictus* is able to establish populations in temperate regions where one of its primary competitors, *Ae*. *aegypti*, cannot withstand the cold climate [5]. *Ae*. *albopictus* is a vector of important arthropod-borne viruses (arboviruses), including dengue (DENV), Zika, and chikungunya virus. In total *Ae*. *albopictus* is capable of transmitting at least 22 different arboviruses [3]. Although its vectorial capacity for DENV is lower than that of *Ae*. *aegypti* [6], *Ae*. *albopictus* has been responsible for dengue epidemics in Japan, Malaysia, China and Hawaii [3]. Moreover *Ae*. *albopictus* has been implicated in the initial spillover of sylvatic dengue virus into the human endemic cycle [7]. *Ae*. *albopictus* is becoming a more important vector for chikungunya virus due to adaptive mutations in emerging strains of the virus [8,9]. The role of *Ae*. *albopictus* in Zika virus transmission remains to be fully characterized [10], but it is clearly a competent vector for this virus [11]. With the recent entry of Zika and chikungunya into the Americas as well as the steady increase in DENV transmission, control of *Ae*. *albopictus* will be critical for curbing of the spread of these viruses, especially in the areas where *Ae*. *aegypti* is absent.

DENV is the most devastating mosquito-borne viral pathogen, causing an estimated 390 million annual infections and almost 100 million cases of dengue disease [12–14]. Dengue disease spans a spectrum of symptoms from mild fever, muscle and joint pain to life-threatening dengue hemorrhagic fever and shock syndrome [15]. DENV comprises four antigenically- and genetically-distinct serotypes [15]. Recently a tetravalent DENV vaccine has been licensed in some DENV-endemic countries, but its use is limited to individuals from 9 to 45 years old [16]. Therefore, control of vector mosquito populations is still a critical component of DENV control. However standard approaches have not been very effective against *Ae*. *aegypti* and *Ae*. *albopictus* [17]. Novel strategies for transmission control are urgently needed, and these must be founded in a detailed knowledge of mosquito-virus interactions.

Cross-talk between *Ae*. *aegypti* and DENV at the molecular level has been extensively studied [18–36]. However, the molecular interactions between *Ae*. *albopictus* and DENV are not as well characterized. Only recent study by Liu *et al*. described differentially expressed transcripts and micro-RNAs (miRNAs) in the midgut of *Ae*. *albopictus* at 1 day following DENV exposure. The authors found that a number of genes and miRNAs were differentially expressed, many of which are implicated in antiviral functions [37]. In the current study we expanded this view of differential replication across time and tissues. Specifically we analyzed the changes in transcriptomes of midguts and midgut-free carcasses *Ae*. *albopictus* in response to DENV-2 infection at one and five days post-infection using next-generation sequencing (NGS). We focused on early time points after DENV infection to reveal *Ae*. *albopictus* genes that respond to midgut invasion and systemic dissemination of DENV. We found that immunity-related genes and genes previously identified as responsive to DENV infection in *Ae*. *aegypti* were also modulated in *Ae*. *albopictus* midgut and carcass. Our study provides insights into important genetic components that respond to DENV infection in this mosquito vector.

## Materials and methods

### Mosquito culture

*Aedes albopictus* (MRA-804) was obtained from MR4 and reared in an environmental chamber kept at 27°C, 80% RH and 16:8 light:dark cycle. Larvae were provided with ground cat food (Special Kitty, Walmart) *ad libitum*. Culture was maintained by feeding the adult females washed erythrocytes from bovine defibrinated blood (Hemostat, Dixon, CA) in 80 mg/mL bovine serum albumin (BSA, Fraction V, Research Products International Corp., Prospect, IL) in 1× phosphate-buffered saline, pH7.4 (PBS, Sigma-Aldrich, St. Louis, IL), supplemented with 1 mM ATP final concentration. Adults used for DENV infection were at least five days post-emergence.

### Dengue virus (DENV)

Dengue serotype 2 New Guinea C strain virus (DENV-2 NGC) was obtained from the Whitehead lab at the National Institutes of Health, passaged in monolayers of C6/36 cells, and a virus stock at a concentration of 7.48 log_10_ plaque-forming units (pfu)/mL was frozen in multiple aliquots at –80°C. *Ae*. *albopictus* C6/36 cells were cultured in Minimum Essential Medium (MEM) supplemented with 10% FBS, 2 mM L-glutamine (Gibco), 2 mM non-essential amino acid (Gibco) and 0.05 mg/mL gentamycin (Invitrogen) and were maintained at 32°C, 80% RH in 5% CO_2_.

### Meal preparation

Two types of bloodmeal were prepared: DENV-2 infected or media control. Bovine red blood cells (RBCs) were separated by centrifugation at 500× g, 4°C for 15–30 min and serum supernatant was removed. The RBCs were washed by adding PBS in equal volume to the RBCs, inverted several times to mix, centrifuged at 500× g, 4°C for 15 min, supernatant PBS was removed, the process was repeated once more and washed RBCs (wRBCs) were resuspended with 80 mg/mL BSA in PBS (pH 7.4) at 50% hematocrit (hereafter wRBC/BSA). For infectious meals, wRBC/BSA and DENV stock or were mixed at 2:1 ratio. For control meals DENV was replaced with C6/36 culture medium. All meals were supplemented with ATP at 1 mM of final concentration as phagostimutant.

### Dengue virus infection

Mosquitoes were transferred to feeding cartons with a mesh top (50–70 females each) one day prior to feeding and starved by removing sugar-soaked pledgets. Sufficient cartons of mosquitoes were prepared to implement four total treatments in this experiment: exposed day 1, control day 1, infected day 5 and control day 5. The term exposed rather than infected is used for day 1 mosquitoes because infection was not determined on this day, however the high levels of infection in our day 5 group suggests that most or all of these day 1 mosquitoes were in fact in the process of becoming infected. Two ml of the designated meal (control or DENV-2) was placed inside a water-jacketed glass feeder with circulating 37°C water with stretched parafilm on the feeding side. On the day of feeding, the starved mosquitoes were acclimated in the feeding facility for at least 30 min and allowed to feed for ~20 min. Aliquots of each meal containing DENV before and after the feeding sessions were collected in Hanks balanced saline solution (HBSS) supplemented with 200 mM sucrose, 3.8 mM of KH_2_PO_4_, 7.8 mM of K_2_HPO_4_ and 6 mM of L-glutamic acid (HBSS/SPG) at meal:HBSS/SPG ratio 1:9. These aliquots were used to verify the virus titer. The average starting DENV concentration was ~7.0 log_10_ pfu/mL, which dropped by approximately 1.0 log_10_ unit by the end of 20 min feeding period. After the feeding, mosquitoes were anaesthetized at –20°C for 2 min, and fully engorged mosquitoes were transferred to clean cartons and kept in environmental chamber at 27°C, 16:8 hr (L:D) cycle, 80% RH, supplied with 10% sucrose ad libitum until frozen on day 1 or day 5 post-feeding. We performed three biological replicates of each treatment using different batches of mosquitoes.

## Sample collection

Mosquitoes were frozen at –20°C overnight prior to dissection of the midguts. Dissection was performed in 1× phosphate-buffered saline (PBS) under stereo-microscope. From fifteen to twenty midguts per replicate from day 1 were pooled in TRIzol (Life Technology, Carlsbad, CA). We did not assess DENV infection on day-1 midgut samples because at this time point residual DENV particles from blood meal were likely to give false positive results. Whole bodies without midgut from day 1 were also pooled and are henceforth termed “carcasses”. For day 5 samples, individual midguts were split into half and stored separately; carcasses were also stored individually. To minimize cross contamination between individuals, forceps, minute probes and dissecting dish used for dissection were treated with 70% ethanol between each mosquito dissection. RNA was isolated from individual half midguts for RT-PCR to detect dengue infection (see below), the remaining half of dengue-positive midguts and carcasses were pooled and used for transcriptome sequencing. From twenty to thirty half midguts were pooled for each replicate. Similar numbers of samples were pooled for uninfected controls for both time points.

### RNA isolation and RT-PCR

RNA was isolated using TRIzol (Life Technologies, Carlsbad, CA) following the product manual. Isolated total RNA was resuspended with 20–50 μL (10 μL for half midguts) of nuclease-free water and stored at –80°C until used. RNA quality and quantity was assessed by a NanoDrop spectrophotometer.

For day 5 individual midgut samples, reverse transcription PCR (RT-PCR) was performed using the Titan One Tube RT-PCR System (Roche, Indianapolis, IN) to screen for infection with DENV. We used 2.5 μL of RNA in 25 μL reaction volume, and reactions were performed at 50°C for 45 min, followed by 94°C for 2 min and 35 cycles of 94°C for 30 sec, 57°C for 30 sec and 68°C for 45 sec, and final extension at 68°C for 8 min. Products were analyzed by agarose gel electrophoresis. DENV-2 NGC-specific primer sequences (D2NGCenv777F and D2NCGenv1265R) are shown in S1 Table.

### Illumina sample preparation and sequencing

Libraries were constructed using Illumina TrueSeq RNA Sample Prep kit (Illumina, San Diego, CA). One microgram (μg) of total RNA for each sample was used as starting material. A total of 30 libraries were generated, including six libraries of midguts and carcasses from unfed mosquitoes. Quality of the libraries were assessed using an Agilent 2100 BioAnalyzer with the High Sensitivity DNA kit (Agilent Technologies, Waldbronn, Germany).

Libraries were normalized prior to loading on the flowcell by qPCR. Thirty libraries were multiplexed into four lanes of Illumina flowcell. Libraries were then sequenced on Illumina HiSeq 2000 instrument to generate 50-nt long single end reads. After sequencing, raw sequence reads were post-processed in order to remove Illumina adapters/primer sequences.

### Reference for mapping and abundance estimation

We used *Ae*. *albopictus* Foshan strain genome assembly, version AaloF1 as reference because none of other references used for preliminary mapping (*Ae*. *aegypti* AaegL3 and *Ae*. *albopictus* transcriptomic assembly generated from adult oocyte, embryo, and larvae [http://albopictusexpression.org/]) resulted in better mapping rate.

### Mapping and differential expression analysis

Post-processed high quality reads for each sample were aligned to the reference using GSNAP [38] (version released on 2014_12_29) (parameters: indel penalty set to 2, maximum mismatches set to 0.06 and all other parameters set to default). Raw read counts were generated using the Alpheus pipeline developed at NCGR [39]. Gene expression was measured as the total number of reads uniquely aligned to the reference per sample and they were binned by annotated gene coordinate. Annotation file in GFF format associated with the assembly in this study was used for gene coordinate information and to bin the reads. Differential expression and related quality control analyses was performed using the Bioconductor package, DESeq [40]. Raw gene read-count values were normalized for differences in library sequencing depth and composition, using methods implemented in DESeq, enabling gene expression comparisons across all samples. Normalization was performed by a scaling method [41]. Differential expression analysis of normalized read counts was assessed with the negative binomial test as implemented in DESeq with the Benjamini-Hochberg false discovery rate (FDR) adjustment applied for multiple testing corrections [42].

Sequencing raw reads were submitted to GenBank SRA database with accession number SRP077936.

### Downstream analysis

We further sorted differential expression data for false discovery rate (FDR) adjusted p-values less than 0.05, for which gene annotation data were downloaded from VectorBase using BioMart tool (http://biomart.vectorbase.org/biomart/martview/3c602c17c667b4fc2a9ddbe703c5c6a8). Significantly modulated genes were manually sorted by the VectorBase gene description and top 10 InterPro domain hits for potential DENV-responsive genes and immunity-related genes based on published literature on *Ae*. *aegypti*, *Ae*. *albopictus* and other invertebrates.

### cDNA synthesis and quantitative real-time PCR (qRT-PCR)

One μg of total RNA from each sample was used to synthesize cDNA. The same RNA samples used to construct Illumina libraries were used. cDNAs were synthesized using SuperScript III Reverse Transcriptase (Life Technologies, Carlsbad, CA), with anchored Oligo d(T)_20_ Primer (Life Technologies). One μg of total RNA, 0.8 μL of anchored Oligo d(T)_20_ (2.5 μg/μL), 1 μL of 10 mM (each concentration) of dNTPs and nuclease-free H_2_O were mixed for a final volume of 13 μL, which was incubated at 65°C for 5 min and on ice for at least 1 min. Then after adding 4 μL of 5× First-Strand Buffer, 1 μL of DTT (0.1 M), 1 μL of RNase OUT (Life Technologies) and 1 μL of SuperScript III Reverse Transcriptase to the mixture, incubated at 50°C for 90 min followed by 70°C for 15 min.

Primers for qRT-PCR were designed using Primer3 server (ver. 4.0.0) [43,44], which amplify 101–131 bp fragments of the cDNA and searched against *Ae*. *albopictus* genome using BLAST tool in VectorBase to exclude potential unspecific amplification. The sequences of the primers are listed in S1 Table. cDNA was diluted 1/50 in nuclease-free H_2_O, of which 5 μL was used for 20-μL reactions using Eppendorf Mastercycler Realplex system (Eppendorf). Reactions were performed with 30 sec at 95°C, followed by 50 cycles of 95°C for 5 sec and 60°C for 20 sec, and melt curve analysis from 50°C to 95°C. Expression was calculated relative to a housekeeping gene (ribosomal protein S7) by the –2^ΔΔCt^ method [45]. Data were analyzed by student’s t-test (unpaired, two-sided).

### Detection of *Wolbachia* infection

As *Ae*. *albopictus* is known to be naturally infected with multiple *Wolbachia* strains [46], we confirmed that the strain of *Ae*. *albopictus* used in this study is infected with *Wolbachia*. We used PCR amplification of *Wolbachia wsp* gene region using specific primer pairs as described in Zhou *et al*. [47]. Briefly, ovaries were dissected in STE buffer (100 mM NaCl, 10 mM Tris-Cl, 1 mM EDTA, pH 8.0) and incubated at 37°C with addition of 0.8 μg/μL (final concentration) of proteinase K for 30 min followed by incubation in boiling water for 5 min. After brief centrifugation, 1 μL of the supernatant was used for PCR reaction (final volume: 25 μL) with 2× Taq Master Mix (NEB). PCR thermal profile was 94°C for 2 min followed by 35 cycles of 94°C for 30 sec, 52°C for 30 sec and 72°C for 1 min, and final extension at 72°C for 10 min. 10 μL of the PCR products were analyzed by agarose gel electrophoresis. Diagnostic primers are described in Zhou *et al*. as well as listed in S1 Table, of which 328F is specific for *w*AlbA, 183F is specific for *w*AlbB, both were in combination with universal reverse primer 691R. This diagnostic PCR was multiplexed containing above three primers in one reaction.

## Results and discussion

This study was designed to identify genes whose expression is up- or down-regulated in *Ae*. *albopictus* infected with dengue virus. We found significant changes in expression of a number of genes when we compared DENV-infected mosquitoes with uninfected controls. Many of these changes likely reflect a suppression of anti-viral response and immunity in *Ae*. *albopictus* mosquitoes similar to that described in *Ae*. *aegypti* after infection with DENV2 (see below for detail).

### DENV infection of *Ae*. *albopictus*

DENV infection in *Ae*. *albopictus* midguts 5 days after feeding for each of the three replicates was 83.3% of 30 midguts, 91.4% of 35 midguts and 75.0% of 40 midguts, and the overall infection rate was 83.0%. Only mosquitoes with DENV-positive midguts were retained for analysis on day 5 post-feeding.

### Sequencing and alignment success

The single-ended (1×50) Illumina sequencing generated total of 763,296,438 reads for the 30 libraries in 4 lanes of Illumina HiSeq2000 flowcell. In average 25,443,214 reads per library (SD 2,381,117 reads) were generated.

Alignment rate using *Ae*. *albopictus* genome (AaloF1) [48] as reference was ~80–85%, i.e., between 20–30 million reads per sample on an average mapped to the *Ae*. *albopictus* genome, with at least 12.9 million reads aligning uniquely. Hence, there was sufficient coverage to map the transcriptomic landscape of this organism in order to detect differentially expressed transcripts.

### Differential expression in the midgut one day after DENV exposure

**Fig 1:**
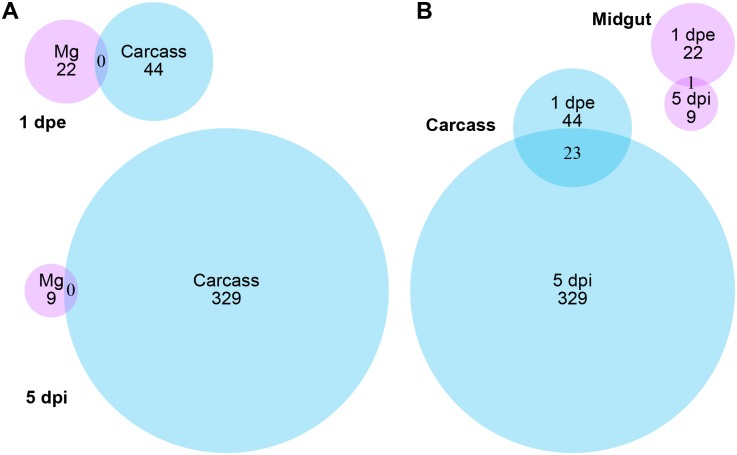
Venn diagrams showing number of significantly modulated *Ae*. *albopictus* genes in the presence of DENV. Pink circles represent midgut, and blue circles represent carcass. A, significantly modulated genes clustered by days post exposure or infection (dpe/dpi), numbers in the middle of circles are numbers of genes, and numbers in overlapped regions are genes overlapped in two categories, Mg: midgut; B, Venn diagram clustered by location.

**Fig 2:**
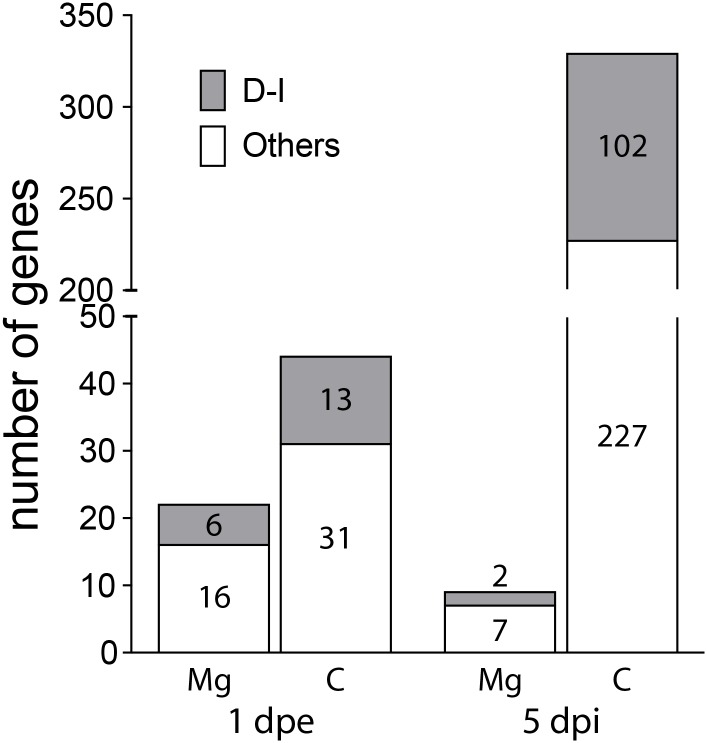
Composition of significantly modulated genes upon DENV infection at 1 dpe and 5 dpi. DENV-responsive and immunity-related genes (D-I) are show as gray bars; other significantly modulated genes are shown as white bars. Numbers of corresponding genes are shown in the bars.

We compared transcript abundance between DENV-infected and -uninfected samples (see Fig 1A). At 1 day after feeding on a DENV-infected blood meal, we found statistically significant changes in expression levels (false discovery rate [FDR]-adjusted p-value < 0.05) in 22 genes in the midgut and 44 genes in the carcass (See S2 and S3 Tables for complete lists of significantly modulated genes) in DENV-exposed compared to control mosquitoes. There was no overlap in the up- and down-regulated genes between midgut and carcass (Fig 1A). Functional classification of these transcripts, based on VectorBase gene descriptions, InterPro domain hits, and literature evidence, suggest that roughly one fourth to one third of them have known functions in DENV-responsive or immunity in *Aedes* mosquitoes and other insects (Fig 2).

**Fig 3:**
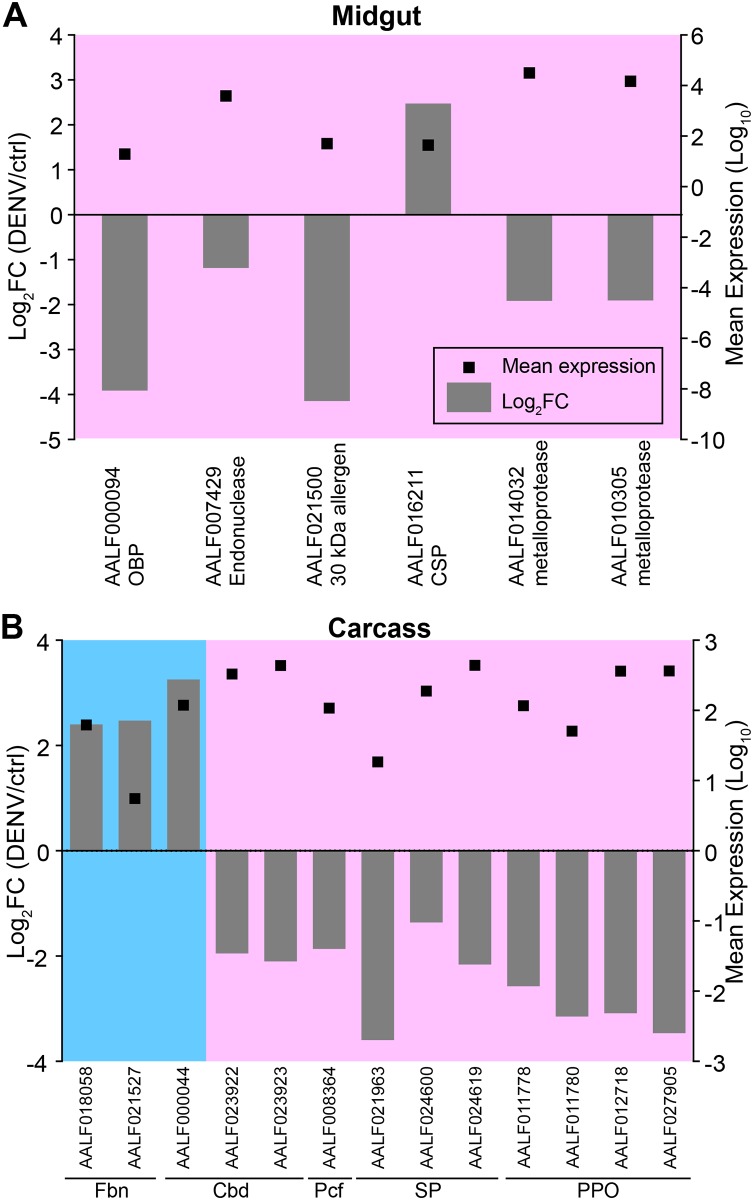
Immunity-related and DENV-responsive transcripts significantly modulated in the presence of DENV in the midgut (A) and carcass (B) at 1 day post exposure (dpe). Grey bars: log_2_ transformed fold change of DENV-exposed relative to control mosquitoes (Log_2_FC[DENV/control]); dots: mean expression levels between exposed and control ([infected + control]/2; Log_10_ scale). Pink backgrounds indicate modulations that we hypothesize, based on the literature, may enhance DENV infection, whereas blue backgrounds indicate modulations that we hypothesize may suppress DENV infection. OBP: odorant binding protein family; CSP: Clip-domain serine protease; Fbn: fibrinogen domain containing family; Cbd: chitin-binding domain containing family; Pcf: pacifastin-like gene; SP: serine protease; PPO: prophenoloxidase.

The only putative immune gene significantly up-regulated in DENV-exposed midguts at 1 day after feeding was a clip-domain serine protease (AALF016211) (Fig 3A). This type of protease has been shown to regulate arbovirus susceptibility in vertebrates as well as vector insects. For example, the presence of an *Ae*. *aegypti* CSP, CLIPA3, expressed in saliva enhances DENV infection in mouse fibroblast cells [49]. Another study showed that experimental inhibition of trypsin-type serine proteases in *Ae*. *aegypti* midguts drastically reduced infection of DENV [50]. The up-regulation of a clip-domain serine protease coinciding with DENV invasion of the *Ae*. *albopictus* midgut suggests a possible involvement of this protease in the infection process. Further studies, for example RNAi knockdown or CRISPR/CAS9-mediated gene knockout, are needed to confirm this hypothesis.

We found transcription of the homologous gene to *Ae*. *aegypti* 30 kDa allergen “aegyptin” down-regulated in midguts of *Ae*. *albopictus* exposed to DENV. Aegyptin has been characterized as an anti-platelet aggregation factor that binds collagen [51]. It is expressed in the salivary glands of *Ae*. *aegypti*, and its expression is down-regulated upon infected with DENV [18,19]. Aegyptin is also known to reduce DENV titers when co-inoculated with DENV in mice [20]. Considering aegyptin’s anti-viral effects in the salivary glands of mosquitoes it is likely to have similar effects in the midgut, but further studies are necessary to confirm this hypothesis and elucidate the mode of action of aegyptin’s antiviral activity.

Two genes encoding putative metalloproteases, AALF014032 and AALF010305, were down-regulated in the midgut upon DENV infection. These genes were annotated as putative immunity genes because the metalloprotease gene family includes angiotensin-converting enzymes (ACEs), which seem to have functions in insect immunity. This is evidenced by the up-regulation of ACEs in insect hemocytes upon stimulation by bacterial component lipopolysaccharide (LPS) [52].

An odorant-binding protein (OBP) family gene was down-regulated in the DENV-exposed midgut. Members of the OBP function as carriers of hydrophobic molecules including odorants and pheromones in the insect antenna. OBP family proteins have also been detected in *An*. *gambiae* hemolymph [53]. They are also suspected to have functions in insect immunity, as evidenced by altered OBP protein levels in immune-challenged *Drosophila melanogaster* [54]. An endonuclease gene that may function as an antiviral molecule, which degrades viral genome, was also down-regulated.

Overall, the modulation of expression of putative DENV-responsive and immune genes in the midgut at 1 day after feeding seems to create favorable conditions for DENV invasion in the *Ae*. *albopictus* midgut. However it must be noted that this hypothesis is based on the literature from other species. Further experiments must be conducted to elucidate the role of these proteins in DENV infection of *Ae*. *albopictus*.

### Differential expression in the carcass one day after DENV exposure

At 1 day after feeding midgut cells are in the process of becoming infected with DENV and the virus has started to replicate, but virus titers within the carcass are very low or non-existent [21]. Therefore changes in gene expression in the carcass upon infection at this time are most likely due to unknown signals that originated in the midgut. Interestingly the transcriptional changes that we found in the carcass at this time point involved twice the number of genes with expression changes than we found in the midgut (see Fig 1A). From the 44 genes identified, three putative immunity genes were up-regulated, while remaining ten were down-regulated (Fig 3B). The three up-regulated genes are putative pattern recognition proteins. One contains a chitin-binding domain, which was implicated in the immune response of *Anopheles gambiae* [55]. The other two encode proteins with fibrinogen C-terminal domains. This family of proteins plays a role in anti-*Plasmodium* responses in *An*. *gambiae* [56], and they have antibacterial properties in other invertebrates [57]. On the other hand, 10 other putative immune genes were down-regulated upon DENV infection. This group includes four prophenoloxidase (PPO) genes, important components of insect immunity [58,59].

### Changes in differential expression between one day post-exposure and five days post-infection

At 5 days post infection (dpi) the blood meal bolus is cleared from the gut. No additional invasion of DENV into the midgut epithelium is expected at this time point, but the infection is disseminating through the remainder of the body, on its way to the salivary glands.

**Fig 4:**
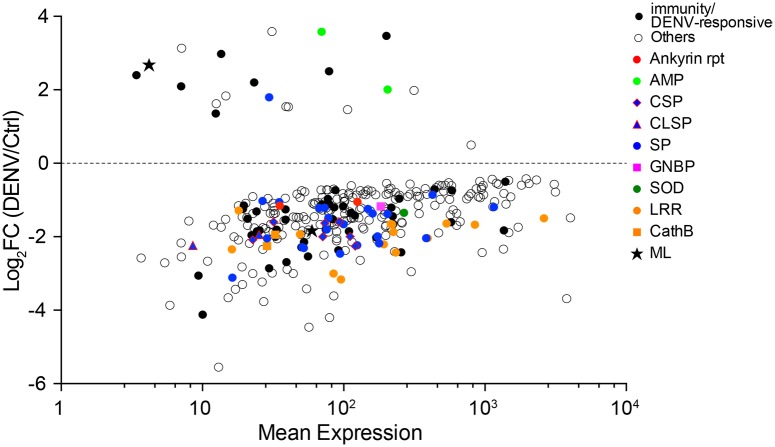
MA plot for significantly modulated genes in DENV-infected *Ae*. *albopictus* carcass five days post-infection in comparison to contemporary, uninfected control. Mean expression level (normalized counts by DESeq) is plotted on horizontal axis; and Log_2_ transformed fold change (Log_2_FC) (DENV/control) is plotted on vertical axis. Filled marks (regardless of shapes) represent genes that have been linked to invertebrate immunity or whose expression was shown to be modulated in DENV-infected *Ae*. *aegypti* or *Ae*. *albopictus*. Red-filled circles: ankyrin repeat domain genes; lime green-filled circles: anti-microbial peptide (cecropin and defensin) genes; blue-filled marks: serine protease genes, for which diamond shapes with red margin are clip-domain containing serine proteases, and triangle shapes with red margin are C-type lectin domain containing serine proteases; pink-filled square: gram negative binding protein (GNBP) gene; dark green-filled circle: superoxide dismutase (SOD) gene; orange-filled circles: leucine-rich repeat (LRR) domain genes; orange-filled squares: cathepsin B genes; star-shaped marks: ML domain genes; black-filled circles: other immunity or DENV-responsive genes; open circles: genes no direct link to immunity or DENV-responsive functions.

Nine and 329 genes were significantly modulated in the midgut and carcass, respectively at 5 dpi (Table 1 and Fig 4). In the midgut, the number of modulated genes at 5 dpi was less than half observed at 1 day post exposure (dpe), but in the carcass the number of modulated genes increased by sevenfold at 5 dpi relative to 1 dpe. This change may reflect direct effects of a disseminated DENV infection of multiple tissues within the carcass, though we did not assay carcasses themselves for infection.

**Table 1:** Transcripts significantly modulated in the presence of DENV in the midgut at 5 days after infection. ^1^VB gene description: VectorBase gene description; ^2^Log_2_FC: Log_2_ transformed fold change DENV-infected versus control. Transcripts with bold letters are immunity-related and DENV-responsive genes.

Gene ID	VB gene description^1^	InterPro domain	^2^Log_2_FC
AALF010748		Lipoprotein_6	Unique in DENV
**AALF000094**		**PBP_GOBP**	**3.91**
**AALF006375**		**Chitin-bd_dom**	**2.71**
AALF028390		Rhs_assc_core	2.69
AALF009141	DNA polymerase epsilon, catalytic subunit	RNaseH-like_dom	2.29
AALF024225	pnuts protein	TFIIS_N	1.66
AALF026259	proliferating cell nuclear antigen	Pr_cel_nuc_antig	1.50
AALF016704	phosrestin i (arrestin b) (arrestin 2)	Arrestin	-1.74
AALF009534	long wavelength sensitive opsin	Opsin	-2.04

### Differential expression in the midgut five days after dengue infection

Out of the 9 genes modulated in the midgut at this time point, 2 were down-regulated and 7 were up-regulated (Table 1). The up-regulated genes include 2 genes that are potentially involved in immunity to DENV. One of them is an OBP gene (AALF000094), which is highly up-regulated at this stage, while it was significantly repressed at 1 day post-exposure. The other gene encodes a protein with chitin-binding domain. These two proteins may be involved in clearing DENV infection from the midgut. While DENV infection persists in the salivary glands for the life of the mosquito, virus titers decline over time in other tissues [60]. We also speculate that these proteins may contribute to interference among arboviruses during multiple arbovirus infection and superinfection exclusion among arboviruses [61].

### Differential expression in the carcass five days after dengue infection

In contrast to the low number of genes modulated in the midgut, 329 genes were significantly modulated in the carcass in the presence of DENV, of which 309 genes were down-regulated and 20 genes were up-regulated (Fig 4 and S5 Table). Roughly one third of these genes are potentially involved in immunity or DENV responsive functions (Fig 2). Below we discuss some of the genes that have been implicated as immune genes based on their domain structure.

#### Ankyrin repeat domain proteins

Two genes with an ankyrin repeat domain were down-regulated in the carcass of Ae. albopictus at 5 dpi (Fig 4, red-filled circles). The Ae. agypti homologue of one of these genes was up-regulated in DENV-infected salivary glands of Ae. aegypti, while silencing of the same gene increased DENV infection [22], suggesting that this particular ankyrin repeat protein has a negative impact on DENV in Aedes mosquitoes. Its down-regulation in the carcass could therefore facilitate the systemic infection and dissemination of DENV.

#### Anti-microbial peptides (AMPs)

Two anti-microbial peptides, a defensin and a cecropin were up-regulated in the carcass 5 dpi (Fig 4, green-filled circles). In *Ae*. *aegypti* several defensin and cecropin transcripts are up- or down-regulated in DENV-infected mosquitoes [23]. Defensin C is upregulated in *Wolbachia*-infected DENV-resistant *Ae*. *aegypti* strains (*w*MelPop-CLA and *w*AlbB). These two strains modulated the expression of a non-overlapping group of AMP genes as well [24,25]. While it is unknown whether these AMPs have any anti-viral effects in mosquitoes, functional characterization of these specific AMPs may reveal anti-DENV effector molecules.

#### Serine proteases

The gene family with the most members modulated in the carcass at 5 dpi are serine-type proteases. Thirty-one transcripts with predicted serine-type protease domain (Fig 4, blue-filled marks) were down-regulated and one up-regulated (AALF022225). Seven of the down-regulated genes are CSPs (clip-domain serine proteases mentioned above; Fig 4, blue-filled diamonds with red margin), and one gene is a late trypsin which plays a critical role in infection, replication and dissemination of DENV in the midgut of Ae. aegypti as inhibition of trypsin drastically reduced DENV titer in the midgut [50]. Two of the down-regulated genes are unique in that they contain both a serine-type protease domain and C-type lectin domain. Such proteins have been shown to function in antifungal defense and melanization cascades active against Plasmodium gallinaceum in Ae. aegypti (Fig 4, blue-filled triangles with red margin) [62,63]. Considering the number of serine protease genes down-regulated significantly (more than 2 fold; Log_2_FC ≤ –1.0, see Fig 4 and S4 Table) we hypothesize that massive down-regulation of this gene family is triggered by the virus in order to suppress the Ae. albopictus systemic immune response.

#### Gram-negative bacteria binding protein (GNBP)

One GNBP was down-regulated in the carcass at 5 dpi (Fig 4, pink square). The homologous gene is involved in the toll pathway in Ae. aegypti. This pathway is important for DENV control in Ae. aegypti [23]. In contrast to our observation, GNBPs are up-regulated in the carcass of DENV-infected Ae. aegypti and interestingly also in Wolbachia wAlbB-infected Ae. aegypti which have become resistant to DENV after infection with the symbiont [26]. The Ae. albopictus strain used in this study is infected with both Wolbachia wAlbA and wAlbB strains (S1 Fig). However, our results show that it is quite permissive to DENV infection, confirming that Wolbachia infections in mosquitoes do not always confer resistance to DENV [25].

#### Superoxide dismutase (SOD)

SOD transcripts are induced in Wolbachia wAlbB-infected Ae. aegypti regardless of DENV infection [26]. One SOD transcript was down-regulated in DENV-infected Ae. albopictus at 5 dpi (Fig 4, green-filled circle). Wolbachia-induced DENV resistance in Ae. aegypti is due to elevated levels of reactive oxygen species (ROS), which coincided with the high levels of antioxidant genes such as SOD [26]. Despite the presence of Wolbachia, Ae. albopictus is not refractory to DENV infection and dissemination ([25] and this study). In fact, Wolbachia infection did not enhance hydrogen peroxide production and antioxidant gene expression in Ae. albopictus [64].

#### Leucine-rich repeat domain proteins (LRR) and cathepsin B

LRRs play a role in the invertebrate immune response. They include toll-like receptors and other pattern recognition molecules [65]. Fourteen transcripts with LRR domain were down-regulated in DENV-infected Ae. albopictus carcass 5 dpi (Fig 4, orange-filled circles). In Ae. aegypti, some LRR genes are enriched in DENV-resistant strains [27]. Down-regulation of 14 LRR transcripts suggests a selective suppression of this type of immune receptor upon DENV infection of Ae. albopictus. Two cathepsin B genes followed the same trend, in that they were down-regulated in DENV-infected Ae. albopictus carcass, but are enriched in DENV-resistant strain of Ae. aegypti.

*MD-2-related lipid-recognition (ML) domain*–One ML domain transcript was down-regulated and another was up-regulated in DENV-infected *Ae*. *albopictus* (Fig 4, filled stars). While silencing of a ML gene in *An*. *gambiae* enhanced O’nyong-nyong virus infection [66], silencing of two proteins with these lipid-recognition domain in *Ae*. *aegypti* resulted in reduction of DENV infection. In *Ae*. *albopictus* these proteins may support DENV infection as in *Ae*. *aegypti* [28].

In summary, transcriptome changes in the *Ae*. *albopictus* midgut at 5 dpi are greatly diminished compared to the 1 dpe time point. However, we observed a drastic increase in the number of significantly modulated genes in the carcass at 5 dpi. About a third of significantly modulated genes can be linked to immunity or were previously identified as DENV-responsive genes in other species of *Aedes* mosquitoes. Specifically the general suppression of many, but not all, of these genes seem to favor DENV infection, if they play the same role in *Ae*. *albopictus* as they do in other mosquitoes.

### Verification of RNA-seq results via qRT-PCR

**Fig 5:**
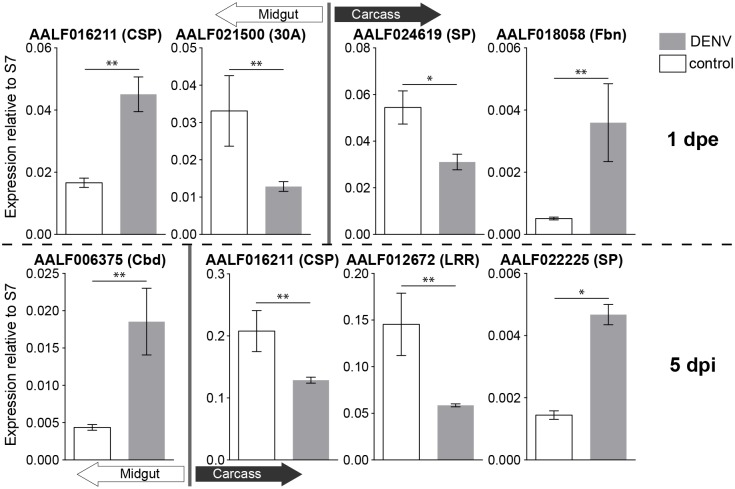
qRT-PCR analysis of DENV-responsive and immunity-related genes. DENV-responsive and immunity-related genes that were significantly up- or down-regulated compared to control by RNA-seq were randomly selected to represent each treatment and time point and relative expression levels were analyzed by qRT-PCR. CSP: clip-domain serine protease; 30A: 30 kDa salivary gland allergen; SP: serine protease; Fbn: fibrinogen-related gene; Cbd: chitin-binding domain containing gene; LRR: leucine-rich repeat gene. DENV: dengue virus-infected samples. Top panels are the genes significantly modulated at 1-day post exposure (1 dpe); bottom panels 5 days post infection (dpi). Expression is shown relative to the expression of reference gene (RPS7) in mean ± SEM. Statistical significance between control and DENV-infected samples is shown with asterisks: *: *p* < 0.05; **: *p* < 0.01.

To verify the results of our RNA-seq analysis, we performed qRT-PCR analysis to compare expression levels of seven immune genes that showed significantly modulated gene expression (Fig 5). In all cases, the modulation of gene expression corresponded with our RNA-seq results (see Figs 3 and 4, Table 1, S2–S5 Tables).

### Summary and insights

While the transcriptional interplay between *Ae*. *aegypti* and DENV during infection has been extensively studied, the response of *Ae*. *albopictus*, which is responsible for recent urban outbreaks of DENV in Japan, Hawaii and elsewhere, has received less attention (but see–[18–23,26–28,34–36,49,50]). To date, the bulk of studies of the molecular cross-talk between *Ae*. *albopictus* and DENV have focused on the impact of host-derived miRNAs—two miRNAs seem to target directly DENV genome sequence, which may be important in controlling DENV in *Ae*. *albopictus* [34,67]. Transcriptomic profiling of *Ae*. *albopictus* miRNAs uncovered miRNAs differentially regulated in response to DENV-2 infection and suggests regulation of immunity genes [68]. A recent study described transcriptomic and miRNA change 1 day following DENV-2 (NGC strain: 10^7^ 50% tissue culture infective dose/mL) infection in the *Ae*. *albopictus* (Foshan strain) midgut and found that a large number of genes (777 transcripts) were significantly modulated unlike our observation (22 genes) [37]. This difference may be due to difference in the strain of *Ae*. *albopictus*, sample preparation methodology, mapping algorism and other factors. We did not include small RNAs like miRNAs in this study, and direct comparison between our study and the studies on miRNAs is not appropriate. Our study took a broader approach, characterizing changes in gene expression in the midgut and the rest of the body (carcass) in *Ae*. *albopictus* across the early stages of DENV infection and dissemination.

**Fig 6:**
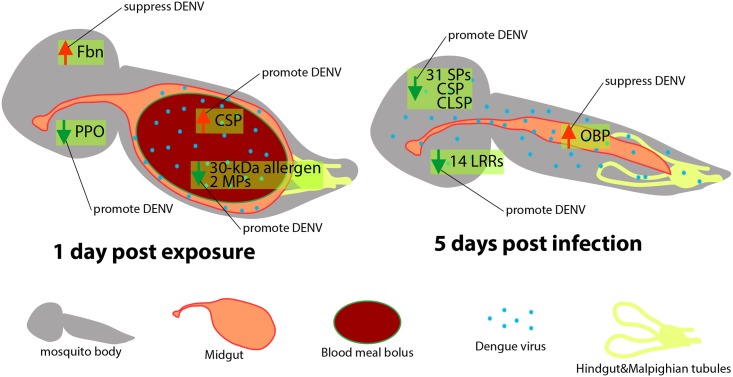
Graphical model of transcriptomic modulation during DENV infection of *Ae*. *albopictus* and its putative impacts on DENV replication. Graphical legends are shown in the figure. The genes with highest number of genes in the same gene family are shown as representatives. Red and green arrows represent up- and down-regulation, respectively, of indicated gene(s) in DENV-infected mosquitoes in comparison to uninfected control. Fbn: fibrinogen domain containing gene; PPO: prophenoloxidase gene; MP: metalloprotease gene; CSP: clip-domain containing serine protease; SP: serine protease; CLSP: C-type lectin containing serine protease; LRR: leucine-rich repeat domain containing gene; OBP: odorant binding protein gene.

The differential gene expression caused during exposure to and infection with DENV was time- and site-specific. On the first day after exposure to DENV, both midgut and carcass showed changes in gene expression, whereas five days post-infection, gene expression in the midgut became more similar to uninfected mosquitoes whereas gene expression in the carcass diverged widely from uninfected mosquitoes (Fig 6). At both time points and in both tissues, gene expression reflected the complexity of host-virus interactions; based on previous literature the bulk of changes seemed to favor DENV infection, but other changes likely reflected mobilization of host immunity. The fact that 83% of the *Ae*. *albopictus* used in this experiment became infected five days after feeding on DENV supports our hypothesis that the transcriptome profile from this species generally reflected changes that facilitate DENV. However, it is well known that different strains of *Ae*. *albopictus* vary in their susceptibility to different strains of DENV [29], and it would be of great interest to extend the current study to probe such variation. In *Ae*. *aegypti* variation in susceptibility to DENV is correlated with basal-level immune activation [27].

The endosymbiont, *Wolbachia* also plays a significant role in DENV susceptibility in *Aedes* mosquitoes. *Ae*. *aegypti* and *Ae*. *albopictus* artificially infected with *Wolbachia* showed significantly reduced DENV infection and dissemination [24,25,31]. However, *Ae*. *albopictus* is naturally infected with two *Wolbachia* strains, while *Ae*. *aegypti* is usually not infected with *Wolbachia* [46,69]. Infection of natural *Wolbachia* (*w*AlbA and *w*AlbB) in *Ae*. *albopictus* has been shown to have no effect on DENV infection and dissemination, but it seems to have an effect on virus invasion in the salivary glands [32,33]. The *Ae*. *albopictus* strain used in the current study is infected with both *w*AlbA and *w*AlbB strains of *Wolbachia* (S1 Fig), making it a useful model to better understand the effects of *Wolbachia* and other factors in conferring resistance to arbovirus infection and dissemination.

In conclusion this study identified genes whose expression is modulated upon DENV infection in *Ae*. *albopictus* in time- and site-specific manner. It lays the foundation to further studies of the genetic and molecular components mosquitoes use to control DENV infection and transmission.

## Supporting information

S1 Fig Diagnostic PCR for *Wolbachia w*AlbA and *w*AlbB strains in *Ae*. *albopictus* strain (MRA-804) used in this study. All three replicates are shown with 100 bp size marker (“M”). Bands at “A” and “B” indicate PCR products specific for *w*AlbA and *w*AlbB, respectively.
(TIF)

S1 Table List of primers used in this study. (XLSX)

S2 Table List of significantly (p < 0.05) modulated genes in the midgut at 1 dpe. 1MgC: control midgut at 1 dpe; 1MgD: DENV-infected midgut at 1 dpe.
(XLSX)

S3 Table List of significantly (p < 0.05) modulated genes in carcass (whole body without midgut) at 1 dpe. 1CC: control carcass at 1 dpe; 1CD: DENV-infected carcass at 1 dpe.
(XLSX)

S4 Table List of significantly (p < 0.05) modulated genes in the midgut at 5 dpi. 5MgC: control midgut at 5 dpi; 5MgD: DENV-infected midgut at 5 dpi.
(XLSX)

S5 Table List of significantly (p < 0.05) modulated genes in carcass (whole body without midgut) at 5 dpi. 5CC: control carcass at 5 dpi; 5CD: DENV-infected carcass at 5 dpi. S2–S4 Tables and S5 Table are formatted in the same manner: Description of columns: GeneID: VectorBase gene ID; Mean Expression: mean of DENV-infected and -uninfected samples post normalization (mean from both conditions (post normalization)). BaseMean (sample name): mean expression of (sample) of triplicated experiments; FC: fold change (DENV-infected/control); Log2FC: log_2_ transformed FC values; pval: p-values; padj: false discovery rate (FDR) adjusted p-values; VB gene description: VectorBase gene description; GO term name: top Gene Ontology term name; InterPro 1–10; top 10 InterPro domain hits. Genes with potential link to immunity and response to DENV infection are shaded with green.
(XLSX)
