# Correction: Dengue virus serotype 2 infection alters midgut and carcass gene expression in the Asian tiger mosquito, *Aedes albopictus*

**DOI:** 10.1371/journal.pone.0192128

**Published:** 2018-01-25

**Authors:** Hitoshi Tsujimoto, Kathryn A. Hanley, Anitha Sundararajan, Nicholas P. Devitt, Faye D. Schilkey, Immo A. Hansen

The primer sequence for qALBrps7F1 in S1 Table is incorrect. The correct sequence is: TTCAATGGTGGTCTGCTGGTTCTT. Please view the correct S1 Table below.

## Supporting information

S1 Table List of primers used in this study. (DOCX)
