# Supplementary figures and images for: Dengue virus serotype 2 infection alters midgut and carcass gene expression in the Asian tiger mosquito, Aedes albopictus
Source: PLoS One. 2017 Feb 2;12(2):e0171345. doi: 10.1371/journal.pone.0171345 (PMC5289563; doi:10.1371/journal.pone.0171345)

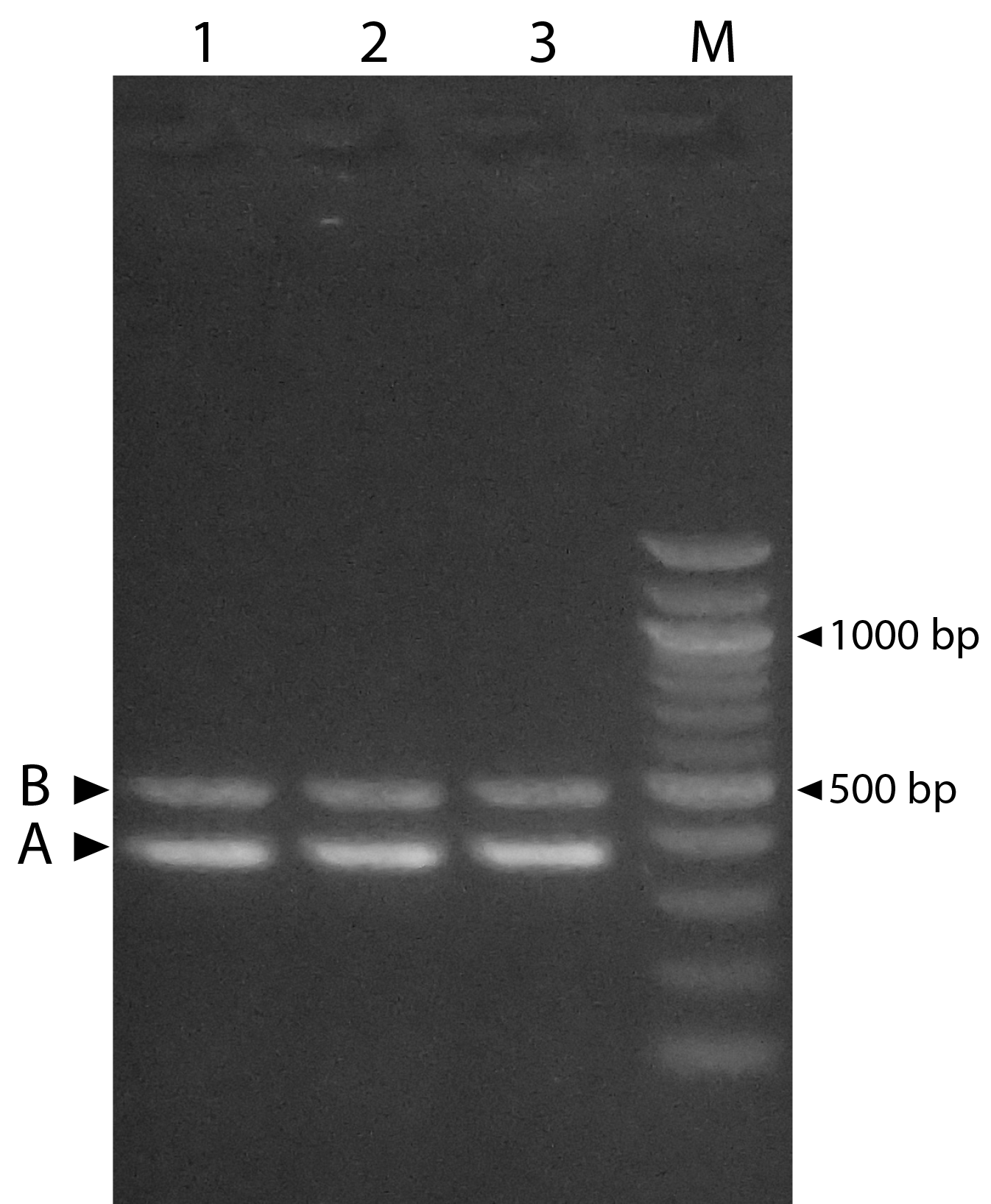

Supplement: S1 Fig — All three replicates are shown with 100 bp size marker (“M”). Bands at “A” and “B” indicate PCR products specific for wAlbA and wAlbB, respectively. (TIF) [file pone.0171345.s001.tif]
