# Supplementary material for: Correction: Dengue virus serotype 2 infection alters midgut and carcass gene expression in the Asian tiger mosquito, Aedes albopictus
Source: PLoS One. 2018 Jan 25;13(1):e0192128. doi: 10.1371/journal.pone.0192128 (PMC5785029; doi:10.1371/journal.pone.0192128)
Supplement: S1 Table — (DOCX) [file pone.0192128.s001.docx]

| **ID** | **Sequence (5'-3')** | **length** | **Note** |
| --- | --- | --- | --- |
| D2NGCenv777F | CATGCACACAGCACTCACAG | 20 | RT-PCR |
| D2NGCenv1265R | AAATCCCAAGCTGTGTCACC | 20 |  |
| qALBrps7F2 | CCGTGTACGATGCCATCCTTGAGG | 24 | qRT-PCR |
| qALBrps7R1 | TTCAATGGTGGTCTGCTGGTTCTT | 24 |  |
| q18058F1 | CAGTGAATGCAACTGGGAGGTCTT | 24 |  |
| q18058R1 | CCGATCCATCTTGTCGCTTCTGAA | 24 |  |
| q24619F1 | CAATCGACCCATGGCACCGACTAG | 24 |  |
| q24619R1 | CCTCCTTGGGTTCGTACATGTTGG | 24 |  |
| q16211F1 | GTCATGGGGCGTAGGATGTGGTAG | 24 |  |
| q16211R1 | AATGCATGTGTAGCTGTTCCTCGT | 24 |  |
| q21500F1 | GTAAGGAATCCGGTGGTTGAAGCT | 24 |  |
| q21500R1 | CGTCAATGACTTTCTTCACGTCGC | 24 |  |
| q12672F1 | GTCATTAGATCGGGGAGCATCACG | 24 |  |
| q12672R1 | AGAGACGTGATACCCAACTGTCCA | 24 |  |
| q22225F1 | GACTGTACACCAACGGACCACTTC | 24 |  |
| q22225R1 | TCCAGCGTTAATTCCAGTTTCCCG | 24 |  |
| q6375F1 | AAGTGCCCACCTAATCTGCATTGG | 24 |  |
| q6375R1 | TGGTTGGTGGAGAAGTTGGTGAAC | 24 |  |
| 183F | AAGGAACCGAAGTTCATG | 18 | *Wolbachia* detection [47] |
| 328F | CCAGCAGATACTATTGCG | 18 |  |
| 691R | AAAAATTAAACGCTACTCCA | 20 |  |
